# IL-36 activates neutrophil extracellular traps and exacerbates LPS-induced ARDS in mice

**DOI:** 10.1038/s41598-026-51329-w

**Published:** 2026-05-09

**Authors:** Haiyan Hu, Lifen Wang, Fu Jin, Shengqin Li

**Affiliations:** 1 Department of Emergency, Zhejiang Provincial Hangzhou Emergency Medical Center, Hangzhou, 310021 Zhejiang China; 2 Emergency and Critical Care Center, Department of Emergency Medicine, Zhejiang Provincial People’s Hospital (Affiliated People’s Hospital), Hangzhou Medical College, Hangzhou, 310000 Zhejiang China

**Keywords:** LPS, ARDS, IL-36, NETs, NF-κB, Diseases, Immunology, Medical research, Pathogenesis

## Abstract

Sepsis-induced acute respiratory distress syndrome (ARDS) is characterized by uncontrolled pulmonary inflammation and neutrophil-driven pathology. Neutrophil extracellular traps (NETs) have been implicated in ARDS progression, but the role of interleukin-36 (IL-36) in this process remains unclear. This study aimed to investigate the regulatory mechanism of IL-36 via NETs and its impact on NF-κB activation in a murine model of LPS-induced ARDS. A lipopolysaccharide (LPS)-induced ARDS model was established in C57BL/6 mice. Animals were divided into Control, ARDS, ARDS + IL-36, and ARDS + IL-36Ra groups. Pulmonary edema was assessed via wet/dry weight ratio, inflammatory cytokines were measured by ELISA, and histopathological changes were evaluated through H&E staining. NETs formation was analyzed using immunofluorescence and Western blot. NF-κB activation was detected via phosphorylation of p65. IL-36 administration exacerbated pulmonary edema, inflammatory cytokine levels (IL-10,TNF-α,MPO) and histopathological injury. Conversely, IL-36 receptor antagonist (IL-36Ra) attenuated these effects. IL-36 promoted NETs formation, as indicated by elevated citrullinated histone H3 (CitH3) and neutrophil elastase (NE) expression. NETs further enhanced IL-36-induced proinflammatory cytokine release and NF-κB activation in bronchial epithelial cells. IL-36Ra partially reversed NET-mediated NF-κB phosphorylation and inflammatory responses. IL-36 exacerbates LPS-induced ARDS by enhancing NETs formation and activating the NF-κB pathway, leading to amplified pulmonary inflammation and injury. Targeting IL-36 signaling may represent a potential therapeutic strategy to mitigate LPS-induced ARDS progression.

**Supplementary Information:**

The online version contains supplementary material available at 10.1038/s41598-026-51329-w.

## Introduction

Acute respiratory distress syndrome (ARDS) is an acute-onset respiratory disorder characterized by non-cardiogenic bilateral pulmonary edema and hypoxemia, resulting from compromised pulmonary endothelial barrier integrity and excessive alveolar-capillary permeability. The pathological hallmarks include diffuse pulmonary inflammation with substantial infiltration of inflammatory cells, predominantly neutrophils and macrophages^1,2^.Clinically, ARDS manifests as bilateral pulmonary infiltrates and respiratory failure^3^, with severity classified as mild, moderate, or severe based on oxygenation parameters^4^.

Epidemiological studies indicate that ARDS occurs in 10% of ICU admissions and 23% of mechanically ventilated patients, with a 28-day mortality rate of 35% that exceeds 40% in severe cases^5^. Sepsis represents the most common predisposing factor for ARDS development, with septic ARDS maintaining mortality rates between 34 and 45%^5,6^, During ARDS progression, neutrophil adhesion, aggregation and activation drive inflammatory cascades through excessive neutrophil extracellular trap (NET) formation^7^. Elevated NETs levels have been consistently documented in LPS-induced ARDS^8–13^. Notably, NETs demonstrate significant correlations with alveolar epithelial/endothelial damage severity and inflammatory mediator concentrations^7,14^, while concurrently releasing proinflammatory cytokines such as IL-1β that exacerbate disease progression^15–17^, However, the precise activation mechanisms underlying these processes remain elusive.

Emerging evidence highlights IL-36/IL-36R signaling as a critical modulator of pulmonary inflammation and immune responses, with constitutive expression throughout lung tissue^18–20^. Bronchial epithelial cells upregulate IL-36 expression following exposure to proinflammatory cytokines and microbial molecular patterns^21^. Experimental models demonstrate that intratracheal IL-36α/γ administration induces proinflammatory cytokine/chemokine production and neutrophil recruitment^22^. The mechanism of IL-36 signaling in sepsis-induced lung inflammation remains unclear. We hypothesize that neutrophil protease-triggered IL-36 activation initiates NF-κB signaling and may regulate septic ARDS via NETs.

## Materials and methods

### Animals

Male C57BL/6 mice (n = 25; aged 6–8 weeks; body weight 22–26 g) were obtained from the Comparative Medicine Center of Yangzhou University (Yangzhou, China). Animals were maintained under specific pathogen-free (SPF) conditions with controlled temperature (25 ± 1 °C), relative humidity (50 ± 10%), and 12–12 h light–dark cycles, with ad libitum access to autoclaved feed and sterile water. All ARDS-related procedures strictly adhered to the National Institutes of Health Guide for the Care and Use of Laboratory Animals and were approved by the Yangzhou University Institutional Animal Care and Use Committee (IACUC Approval No. 202406021).

### Sepsis model

**Fig. 1:**
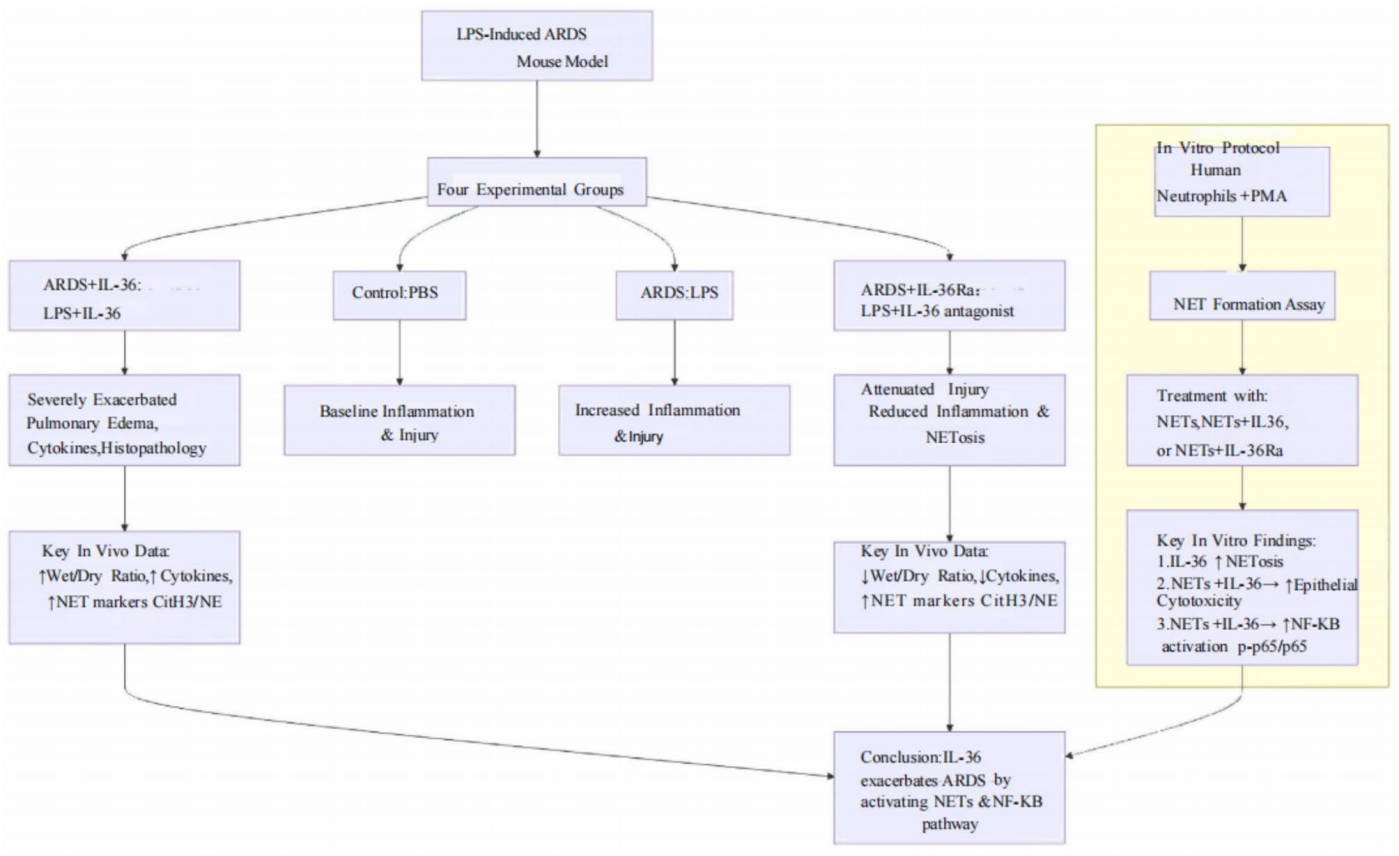
This graphical abstract summarizes the experimental design and main findings. In vivo, an LPS-induced ARDS model in mice demonstrated that exogenous IL-36 worsened pulmonary edema, inflammation, and histopathological injury, while IL-36Ra attenuated these effects. IL-36 promoted NET formation, indicated by elevated CitH3 and NE. In vitro, IL-36 potentiated PMA-induced NETosis in human neutrophils. Co-treatment of bronchial epithelial cells with NETs and IL-36 led to increased cytotoxicity and NF-κB activation, establishing a pro-inflammatory feedback loop.

Mice were randomly assigned to experimental groups using a computer-generated random number sequence divided into Control, ARDS , ARDS + IL-36, ARDS + IL-36 Ra(as illustrated in Fig. 1). To induce LPS-induced ARDS, Mice were anesthetized by intraperitoneal injection of ketamine (87.5 mg/kg) and xylazine (12.5 mg/kg) in sterile PBS. Depth of anesthesia was monitored by absence of pedal reflex.intratracheal instillation of LPS was performed (10 mg/kg^23^, diluted to 100ul with PBS, Cat. No: S1732, Beyotime, China), The source of LPS was Escherichia coli (E. coli) serotype O111:B4,To facilitate LPS distribution throughout the lungs, the mice were mechanically ventilated using a 20-gauge tracheal intubation and animal ventilator (ALCBIO, China) in volumetric control mode. The mice in the PBS group were given the same amount of PBS without LPS by intratracheal instillation in the same way.The ARDS + IL-36group received intradermal injection of 1 μg (diluted to 20 μL PBS) of IL-36^24^ (IL-1F9, Cat. NO:HY-P72544, MCE, USA) 24 h before LPS challenge,The ARDS + IL-36Ra group received intradermal injection of 1 μg (diluted to 20 μL PBS) of IL-36 receptor antagonist(IL-1F5,Cat. NO:HY-P77007, MCE, USA) 24 h before LPS challenge;The following parameters were applied: 7 mL/kg body weight, 120 breaths/minute, positive end-expiratory pressure (PEEP) of 2 cm H_2_O for 10 min. Following ventilation, the mice were monitored for the subsequent 24 h in an animal care facility.All mice were euthanized 24 h post-LPS challenge by cervical dislocation under deep anesthesia.

### Pulmonary edema quantification

Lung edema was evaluated using the wet-to-dry weight ratio method. Fresh right lung tissues were weighed for wet weight and desiccated at 80 °C under vacuum until constant dry weight was attained. Triplicate biological replicates were measured using an analytical balance (± 0.1 mg precision). Th lung was calculated as (W/D) × 100%, effectively eliminating interference from residual blood in tissue hydration assessment.

### Histopathological evaluation of lung tissues

Left lungs were fixed in 4% paraformaldehyde were paraffin-embedded and sectioned into 5 μm slices for H&E staining.Lung injury was assessed using the Smith scoring method,Histopathological scoring was performed by two blinded investigators using a semi-quantitative scale (0–4) for alveolar congestion, leukocyte infiltration, septal thickening, and intra-alveolar hemorrhage.The scoring criteria were as follows: 0 points = no injury; 1 point = lesions involving < 25% of the field; 2 points = 25–50% involvement; 3 points = 50–75% involvement; and 4 points = diffuse involvement throughout the entire field. The total lung injury score was calculated by summing the scores from all categories. For each animal, ten high‑power fields were examined, and the average score was recorded.Microscopic images were acquired using a Nikon Eclipse Ni-U system with DS-Ri2 camera, enabling structural validation.Inter-observer reliability for the semi-quantitative scores was assessed using the intraclass correlation coefficient (ICC), confirming excellent agreement (ICC > 0.85).

### Neutrophil isolation and NETs analysis

Human neutrophils were isolated from peripheral blood of healthy volunteers. The study was approved by the Ethics Committee of Zhejiang Provincial People’s Hospital, and written informed consent was obtained from five participants.Human fresh anticoagulated blood was combined with saline and sedimentation solution (Cat. No: R1010, Solarbio,China) in a 1:1:1 ratio to facilitate the removal of erythrocytes. After sedimentation, the leukocyte-rich supernatant was layered onto a density gradient medium (Reagent A/C) and centrifuged (600–1000 × g, 25–30 min) to isolate neutrophils (Cat. NO: P9040,Solarbio,China). Cells were washed, resuspended in RPMI 1640(Cat. No: C11875500BT, Gibco) medium, and stimulated with 100 nM PMA(Cat. No:HY-18739,MCE,USA) at 37℃ and in a 5% CO2 incubator to induce NETs formation.The NETs layer was collected, homogenized, and centrifuged to remove debris. DNA content was quantified using the Quant-iT™ PicoGreen® assay (Cat. NO: P11496,Thermo,USA), Fluorescence was measured at an excitation wavelength of 480 nm and an emission wavelength of 520 nm.

### NETs viability by trypan blue exclusion

Isolated NETs were assessed for viability using trypan blue exclusion. A 4% trypan blue stock was prepared by dissolving 4 g powder in distilled water, grinding, adjusting to 100 mL with ddH2O, filtering, and storing at 4 °C. The stock was diluted to 0.4% with PBS before use. Cells were trypsinized to a single-cell suspension, diluted appropriately, and mixed with 0.4% trypan blue at a 9:1 ratio. Viable (unstained) and non-viable (blue-stained) cells were counted within 3 min using a haemocytometer.

### Cell culture and treatment

The human bronchial epithelial cell line BEAS-2B was purchased from the Cellverse Co., Ltd(Cat. NO:iCell-h023). Cells were cultured in DMEM(Cat. No: C11885500BT, Gibco) medium containing 10% fetal bovine serum, 100 μg/ mL streptomycin and 100 U/mL penicillin at 37℃ and in 5% CO2 incubator.NETs were incubated with Cathepsin G inhibitor (5uM,Cat. No:HY-103351, MCE,USA) or elastase inhibitor (5 μM,Cat. No: HY-N4331, MCE,USA )for 30 min. Subsequently, IL-36α, IL-36β, or IL-36γ (50 nM) in 1 mL HBSS/0.25% BSA were added to NETs and incubated at 37 ℃ for 30 min. The resulting conditioned supernatants were collected.For co-culture experiments, BEAS-2B cells or isolated neutrophils were treated for 30 min under the following conditions: control (fresh medium), NETs, NETs + IL-36, NETs + IL-36Ra.

### Enzyme-Linked Immunosorbent Assay (ELISA)

Whole blood collected via orbital bleeding from mice was clotted (RT, 1–2 h), centrifuged (3000 × g, 10 min), and serum stored at -80 °C for subsequent analysis. Post-bleeding, bronchoalveolar lavage fluid (BALF) was obtained by saline instillation (3 × 3 mL), centrifuged (3000 × g, 15 min, 4 °C), and the supernatant analyzed for total protein (BCA assay) and cytokines. Serum and BALF levels of MPO (Mlbio,Cat. NO:m l002070), TNF-α (Mlbio, Cat. NO:ml 002095), and IL-10 (Mlbio, Cat. NO:m l037873) were quantified by ELISA using analyte-specific sample dilutions. Separately, Neutrophil cells were cultured in RPMI 1640/10% FBS; PMA-stimulated neutrophils, pretreated with Cathepsin G/Elastase inhibitors (5 μM), were incubated with IL-36 isoforms (50 nM), and the resultant supernatants applied to NETs to assess TNF-α and IL-10 production by ELISA.

### Immunofluorescence staining

Neutrophils were seeded onto glass-bottomed 12-well plates (NEST) at 40–50% confluency and cultured for 24 h (37 °C, 5% CO₂). Cells were fixed with 4% paraformaldehyde (PFA; Sigma-Aldrich,Germany) for 20 min at 25 °C, permeabilized with 1% Triton X-100 (Sigma-Aldrich,Germany) for 5 min, and blocked with 5% goat serum (Gibco) for 30 min. Primary antibodies targeting citrullinated histone H3 (H3Cit, 1:200,Cat. NO:ab5103, Abcam,China) and myeloperoxidase (MPO, 1:100,Cat. NO:ab90810, Abcam,China ) were applied overnight at 4 °C. After three PBS washes, slides were incubated with species-specific secondary antibodies. Goat anti-Mouse IgG-Alexa Fluor® 647 (1:200,Cat. NO:ab150115, Abcam,China ) and Goat anti-Rabbit IgG-Alexa Fluor® 488 (1:200,Cat. NO:ab150077, Abcam,China) for 1 h at 25 °C protected from light. Nuclei were counterstained with 1 μg/mL DAPI (Sigma-Aldrich) for 5 min. Images were acquired using a Leica TCS SP8 confocal microscope with 63 × oil immersion objective (LAS X software), and fluorescence intensity was quantified using ImageJ.

### Cell Viability Assays (CCK-8)

Neutrophil cells were treated with NETs, IL-36, or IL-36Ra for 30 min.After 24 h, 10 µL of CCK-8 reagent(Cat. NO:C 0037,Beyotime,China)was added to each well.The plate was incubated for 2 h, Absorbance was measured at 450 nm using a microplate reader.

### Western Blot

NE, CitH3, p-p65 NF-κB, and p65 NF-κB expression were analyzed. Protein extraction. Lung tissue was homogenized in lysis buffer and centrifuged (12,000 rpm, 4 °C, 10 min). Bronchial epithelial cells were lysed in RIPA buffer with Protease Inhibitor Cocktail Set III (Animal-free, EDTA-Free, Cat. No:HY-K0010, MCE,USA) and centrifuged. Protein concentration was determined by BCA assay(Cat. No:P0012, Beyotime, China) .SDS-PAGE. Samples (25 µg/lane) were denatured, loaded onto 10% gels, and electrophoresed. Proteins were transferred to PVDF membranes at 65 V for 2 h. Membranes were blocked (1 h, RT), then incubated overnight at 4 °C with .NE (1:500,Cat. NO:AF0010,Affinity,China),CitH3 ( 1 µg/mL,Cat.NO.ab5103,Abcam,China ),p-p65 NF-κB ( 1:500,Cat. NO:AF2006,Affinity,China),p65 NF-κB ( 1:500,Cat.NO:AF5006,Affinity,China ),GAPDH ( Cat. NO:ab245355,Abcam,China).After TBST washes, membranes were incubated with species-appropriate horseradish peroxidase (HRP)-conjugated secondary antibodies (1:5000 dilution) for 1 h at room temperature. Protein bands were visualized using an enhanced chemiluminescence (ECL) detection kit (Beyotime) and imaged with a chemiluminescence imaging system. Quantification. Band intensity ratios (target/GAPDH) were analyzed.

### Statistical analysis

The sample size for each experimental group was determined based on the effect sizes and variance reported in prior literature employing analogous murine models of LPS-induced ARDS, supplemented by internal pilot data. Although a formal a priori power calculation was not performed for this exploratory study, future confirmatory investigations will include prospective power analysis. Normality was assessed using Shapiro‑Wilk tests; non‑normally distributed data were confirmed by non‑parametric methods (Kruskal‑Wallis/Mann‑Whitney U) with consistent results. All quantitative data are presented as mean ± standard deviation (SD). The number of independent biological replicates (n) is explicitly indicated in the respective figure legends. Statistical differences between groups were assessed using one-way ANOVA followed by Tukey’s post-hoc test, with a *p*-value < 0.05 considered statistically significant.

## Result

### IL-36 exacerbates pulmonary edema, inflammation, and injury in murine ARDS

**Fig. 2:**
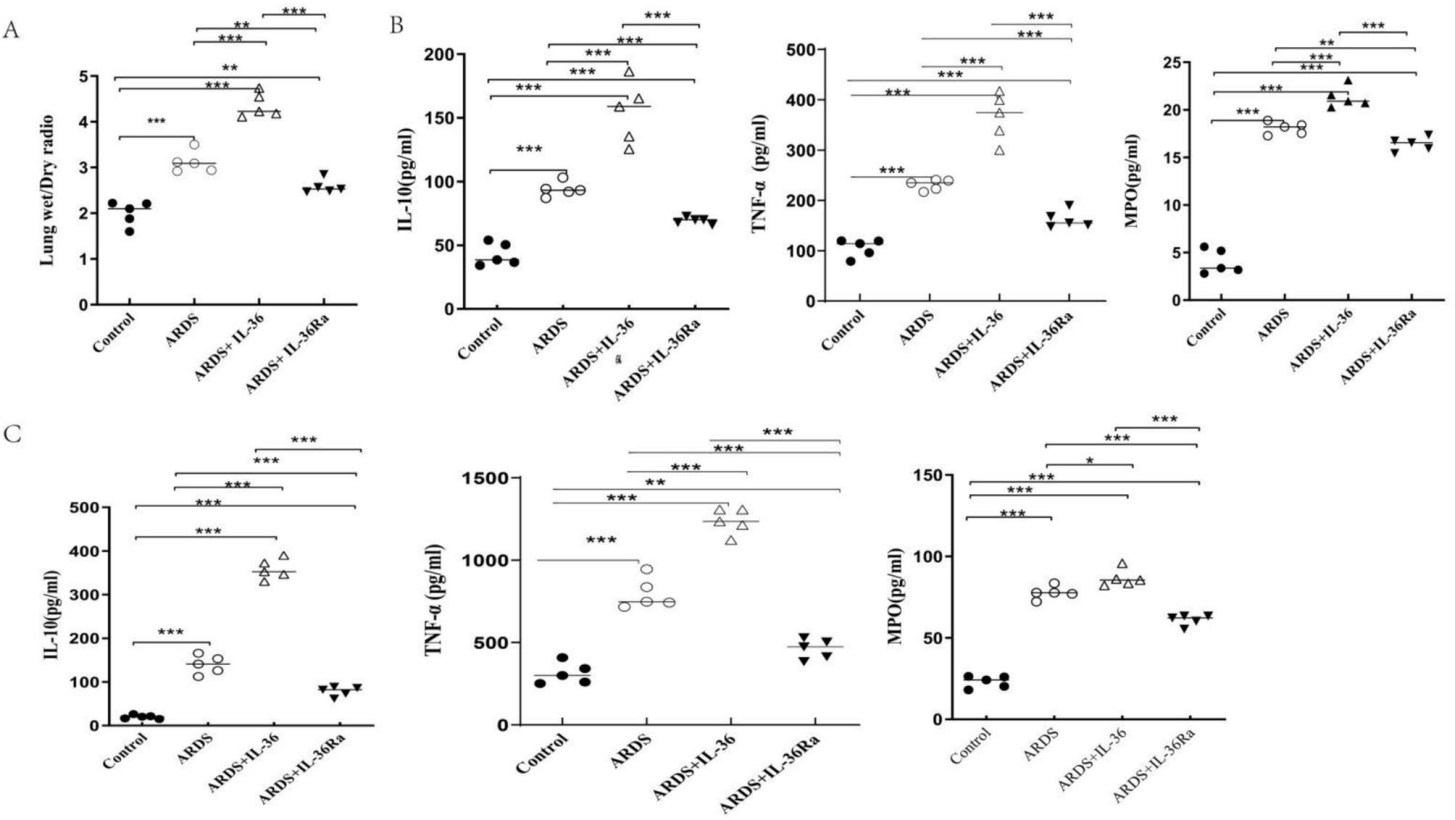
IL-36 exacerbates pulmonary edema and inflammation in LPS-induced ARDS. (**A**–**C**) C57BL/6 mice were pre-treated intradermally with IL-36 (1 µg) or IL-36Ra (1 µg) or vehicle (PBS) 24 h prior to intratracheal instillation of LPS (10 mg/kg) or PBS (Control). Assessments were performed 24 h post-LPS. (**A**) Lung wet-to-dry weight (W/D) ratio as a measure of pulmonary edema (n = 10 per group from two independent cohorts). (**B**) Levels of the inflammatory mediators TNF-α, IL-10, MPO, and total protein in bronchoalveolar lavage fluid (BALF), quantified by ELISA and BCA assay (n = 5). (**C**) Serum levels of TNF-α, IL-10, and MPO quantified by ELISA (n = 5). Data are presented as individual data points with mean ± SD. *p < 0.05, **p < 0.01, ***p < 0.001 by one-way ANOVA with Tukey’s post-hoc test.

The lung wet-to-dry weight ratio exhibited a progressive elevation across the experimental groups. The control group demonstrated the lowest value (2.00 ± 0.24), followed by the ARDS with IL-36Ra group (2.58 ± 0.14), then the ARDS group (3.11 ± 0.21), with the ARDS with IL-36 group showing the highest ratio (4.36 ± 0.24) (Fig. 2A). This escalating pulmonary edema correlated with a commensurate increase in inflammatory mediators. The levels of myeloperoxidase (MPO), TNF-α, and IL-10 in both bronchoalveolar lavage fluid (BALF, Fig. 2B) and serum (Fig. 2C) displayed an identical ascending order from control to ARDS with IL-36Ra to ARDS to ARDS with IL-36, with all pairwise comparisons being statistically significant (*p* < 0.05). Consequently, IL-36 receptor blockade via IL-36Ra mitigated pathological injury, while exogenous IL-36 administration exacerbated ARDS manifestations in a dose-dependent manner, highlighting the central pathogenic role of the IL-36 pathway.

### Histopathological assessment reveals IL-36 exacerbates lung injury.

**Fig. 3:**
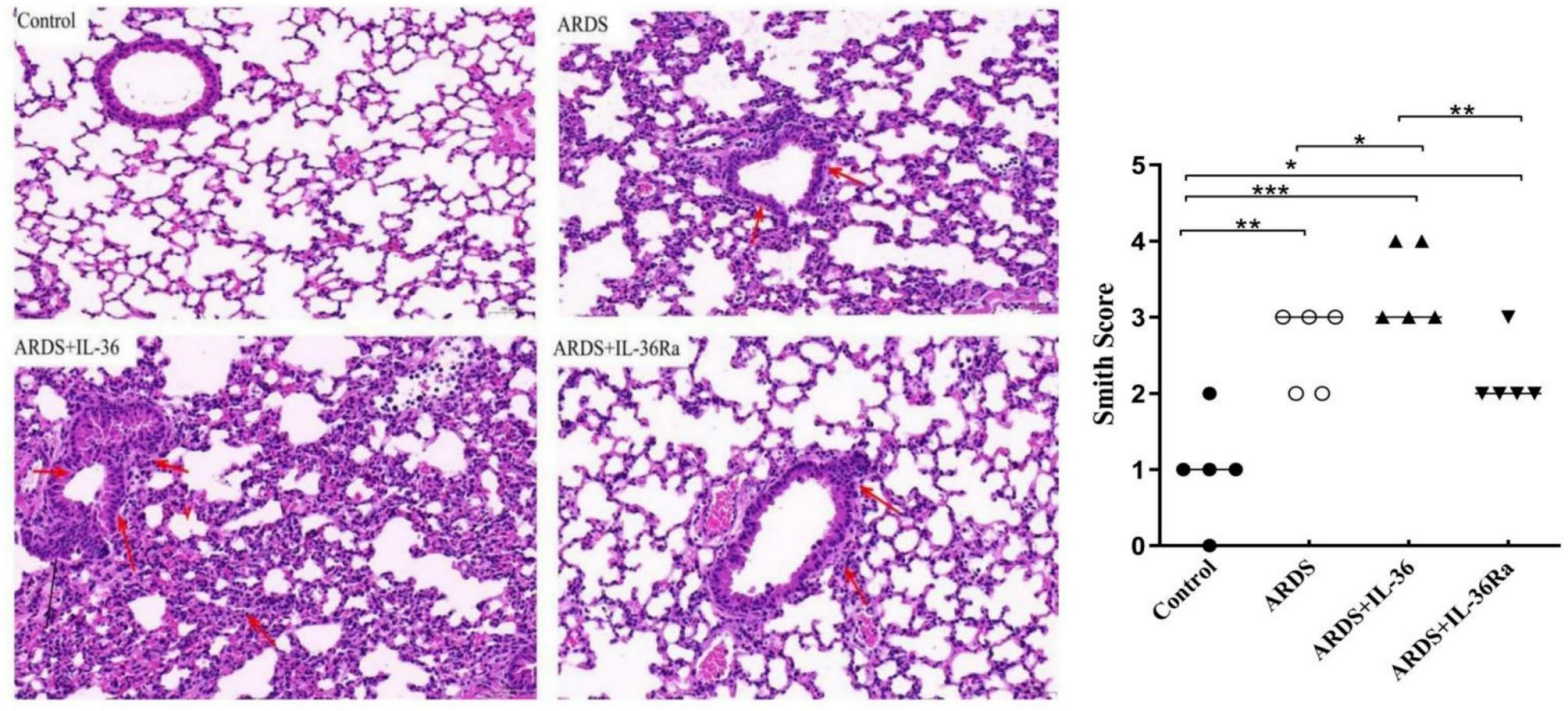
IL-36 augments histopathological lung injury.Representative hematoxylin and eosin (H&E)-stained lung sections from mice in the indicated treatment groups (as in Fig. 1), harvested 24 h post-LPS/PBS. Images show. Control lungs with normal architecture; ARDS + IL-36Ra and ARDS groups with mild edema and inflammation; ARDS + IL-36 group exhibiting severe alveolar collapse, interstitial edema, alveolar wall thickening, and robust inflammatory cell infiltration. Red arrows indicate sites of inflammatory cell infiltration. Scale bar = 50 µm (n = 5).Data are presented as individual data points with mean ± SD. *p < 0.05, **p < 0.01, ***p < 0.001 by one-way ANOVA with Tukey’s post-hoc test..

H&E staining demonstrated progressive histological damage across groups (Fig. 3). Control lungs exhibited intact alveolar architecture, absent hemorrhage, edema, or inflammatory infiltration. Both ARDS + IL-36Ra and ARDS groups displayed mild interstitial edema, minimal alveolar wall thickening, and sparse inflammatory cell infiltration. In contrast, ARDS + IL-36 mice manifested severe pathology:alveolar collapse, pronounced interstitial edema, marked alveolar wall thickening, and robust inflammatory cell, It is consistent with the score result of Smith (Fig. 3).

### IL-36 drives NETs formation and neutrophilic inflammation in ARDS lungs

**Fig. 4:**
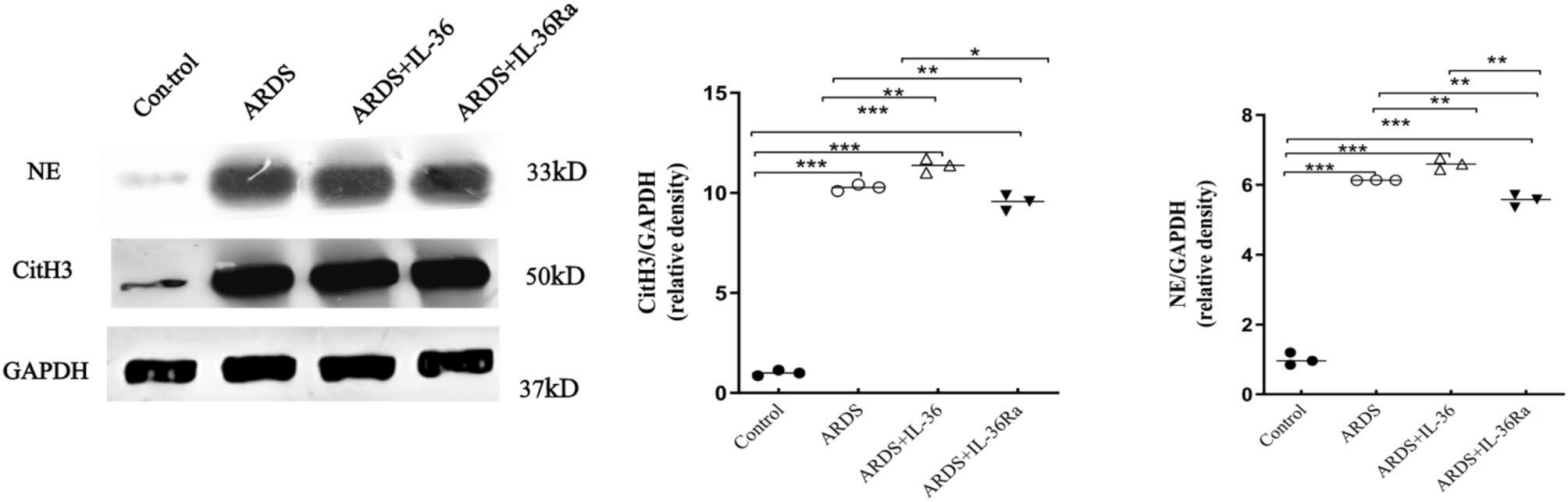
IL-36 signaling promotes the expression of NETosis markers in injured lungs. Representative Western blots of citrullinated histone H3 (CitH3) and neutrophil elastase (NE) in lung tissue homogenates from the indicated treatment groups (as in Fig. 1). GAPDH served as the loading control.Densitometric quantification of CitH3 and NE protein levels normalized to GAPDH (n = 5 per group). Data are presented as individual data points with mean ± SD. *p < 0.05, **p < 0.01, ***p < 0.001 by one-way ANOVA with Tukey’s post-hoc test.

Western blot analysis demonstrated a progressive increase in the expression of neutrophil elastase (NE) and citrullinated histone H3 (CitH3),both established markers of NETosis-across the experimental groups, with the lowest expression in the Control group, followed by the ARDS + IL-36Ra group, then the ARDS group, and the highest expression in the ARDS + IL-36 group. This IL-36 dependent upregulation of NETosis-associated proteins correlated with graded elevations in myeloperoxidase (MPO), pulmonary edema, and proinflammatory cytokine levels, suggesting that dysregulated neutrophil activation represents a central mechanism underlying IL-36-aggravated ARDS (Fig. 4).

### IL-36 potentiates neutrophil NETosis, driving ARDS pathology

**Fig. 5:**
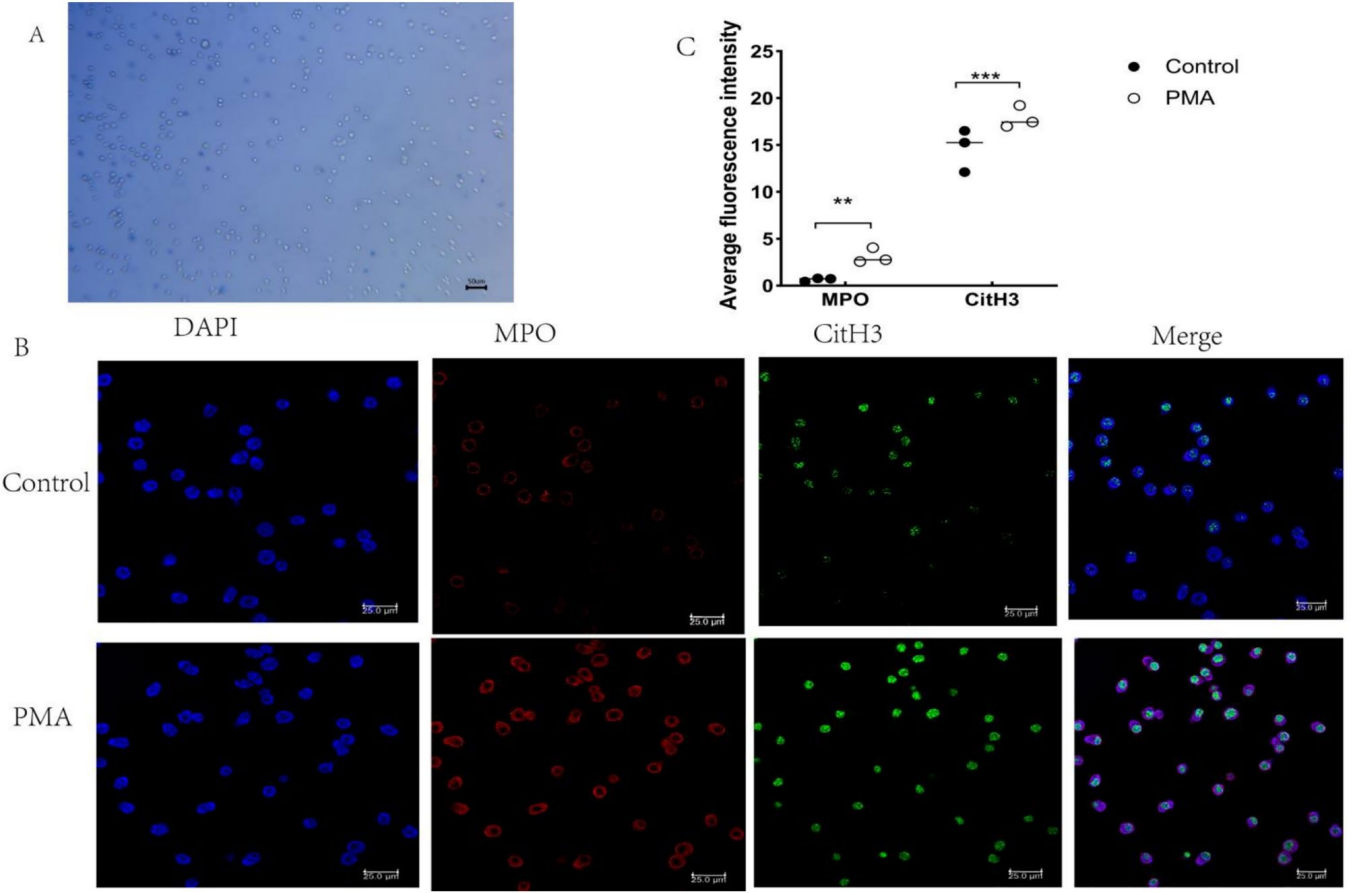
IL-36 primes human neutrophils for enhanced NETosis. (**A**) Viability of freshly isolated human neutrophils assessed by trypan blue exclusion assay prior to experiments. Viable cells (unstained, red arrows) exclude the dye; non-viable cells (blue-stained, black arrows) take it up. (**B**) Representative immunofluorescence images of neutrophils stimulated with PMA (100 nM, 4 h) following pre-treatment with or without IL-36 (50 nM, 30 min). Cells were stained for myeloperoxidase (MPO, red), citrullinated histone H3 (CitH3, green), and nuclei (DAPI, blue). Co-localization (yellow) indicates NETosis. Scale bar = 20 µm. (**C**) Quantification of the mean fluorescence intensity for MPO and CitH3 from images as in (**B**) (n = 3 independent experiments). Data are presented as individual data points with mean ± SD. **p < 0.01, ***p < 0.001 by unpaired two-tailed t-test.

After isolation of human neutrophils using a commercial kit and assessment of viability by trypan blue exclusion, we first confirmed that the isolated cell population was highly viable (> 98%) by trypan blue exclusion assay (Fig. 5A), ensuring that subsequent functional responses were not confounded by baseline cell death. Viable neutrophils primed with IL-36 exhibited an enhanced responsiveness to PMA stimulation. Immunofluorescence analysis demonstrated increased co-localization of MPO and CitH3, indicative of elevated neutrophil extracellular trap (NETs) formation, compared with control conditions. These results provide mechanistic evidence linking IL-36 to enhanced neutrophil-driven inflammatory responses in ARDS (Fig. 5B,C).

### NET-associated IL-36 signaling drives epithelial cytotoxicity while NETs proteases amplify IL-36-dependent inflammation

**Fig. 6:**
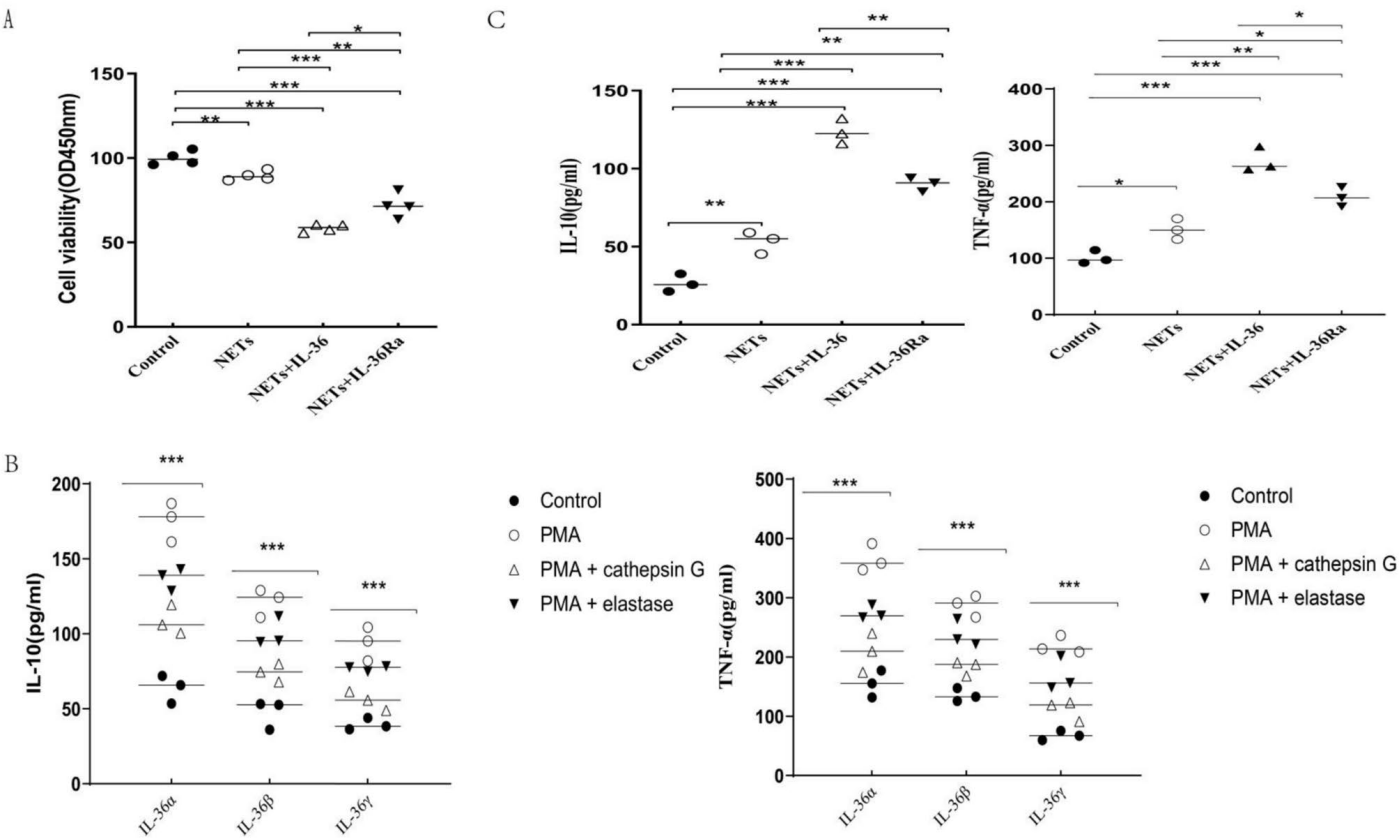
NET-associated IL-36 signaling drives epithelial cytotoxicity and inflammatory cytokine release. (**A**) Viability of BEAS-2B bronchial epithelial cells assessed by CCK-8 assay after 24-h treatment with NETs, IL-36, or IL-36Ra as indicated (n = 3); (**B**) Levels of TNF-α and IL-10 in supernatants from PMA-stimulated neutrophils pre-treated with elastase inhibitor (EI), cathepsin G inhibitor (CGI), or vehicle, followed by incubation with different IL-36 isoforms (IL-36α, β, γ), quantified by ELISA (n = 3); (**C**) Levels of TNF-α and IL-10 in supernatants from BEAS-2B cells treated with NETs in combination with IL-36 or IL-36Ra, quantified by ELISA (n = 3).Data are presented as individual data points with mean ± SD. *p < 0.05, **p < 0.01, ***p < 0.001 by one-way ANOVA with Tukey’s post-hoc test.

CCK-8 assays showed reduced 24-h cell viability, with the control exhibiting the highest viability, followed by NETs, NETs + IL-36Ra, and NETs + IL-36 (Fig. 6A). ELISA of supernatants from IL-36α/β/γ-stimulated cells indicated that PMA most strongly enhanced TNF-α and IL-10 release, followed by PMA with elastase, then PMA with cathepsin G, and lastly the control (Fig. 6B). Similarly, NET-associated proteases hierarchically enhanced PMA-induced cytokine production. Lowest in control, higher with NETs, further elevated with NETs + IL-36Ra, and highest with NETs + IL-36 (Fig. 6C). These findings suggest NETs form a self-amplifying inflammatory circuit whereby proteases increase IL-36 expression, and IL-36 augments NET-mediated cytotoxicity.

### NETs trigger sustained NF-κB activation in bronchial epithelium via IL-36 signaling

**Fig. 7:**
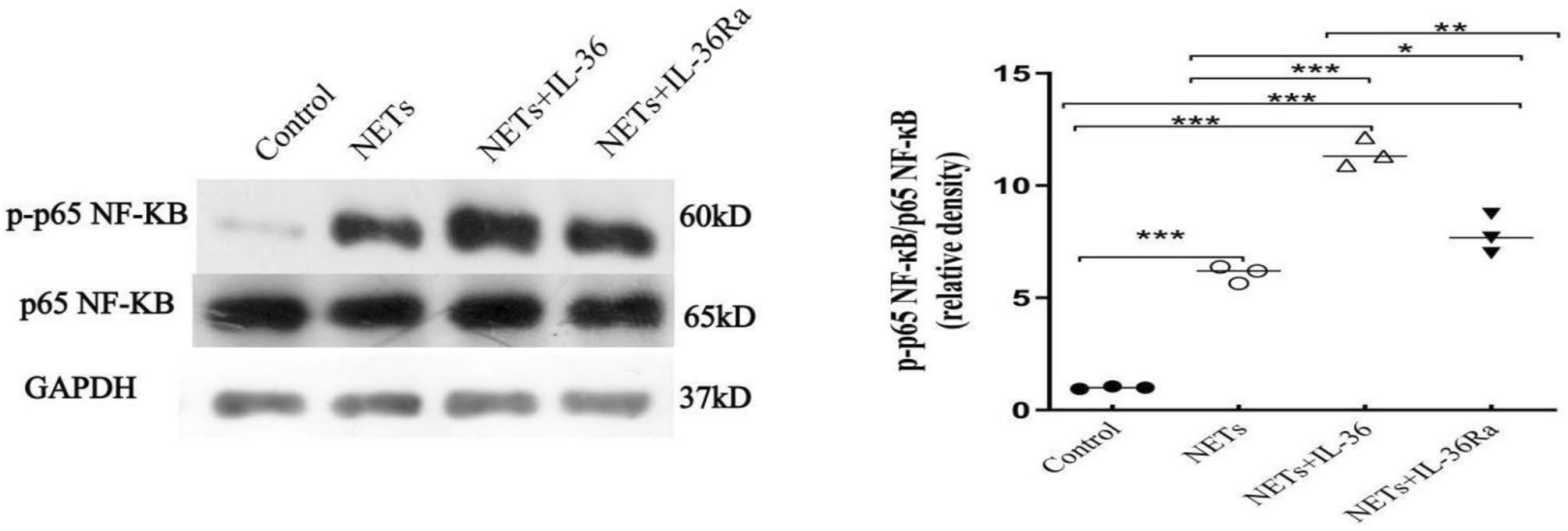
IL-36 amplifies NET-induced NF-κB activation in bronchial epithelial cells. (**A**) Representative Western blot of phosphorylated p65 (p-p65) and total p65 in BEAS-2B cell lysates following 30-min treatments as indicated. (B) Densitometric quantification of the p-p65/p65 ratio (n = 3 independent experiments). Data are presented as individual data points with mean ± SD. *p < 0.05, **p < 0.01 by one-way ANOVA with Tukey’s post-hoc test.

Western blot analysis of nuclear fractions demonstrated a progressive increase in p65 NF-κB phosphorylation across experimental conditions. The lowest p-p65/p65 ratio was observed in the control group, followed by NETs-treated samples, then NETs co-treated with IL-36Ra, and finally NETs with IL-36 showing the highest phosphorylation level (Fig. 7). This IL-36-mediated amplification of p65 phosphorylation identifies NF-κB as a central transcriptional mechanism underlying NET-induced epithelial inflammation. Notably, IL-36Ra attenuated but did not completely abolish NF-κB activation.

## Discussion

As a member of the IL-1 cytokine family, IL-36 shares conserved β-trefoil fold structure and signaling features with other family members such as IL-1α, IL-1β, IL-18, and IL-33, and activates NF-κB and MAPK pathways via the common co-receptor IL-1RAcP^25^. However, unlike IL-1βwhich is predominantly produced by myeloid cells, IL-36 (α, β, γ) is constitutively expressed in barrier tissues including the lung, skin, and intestine, and is rapidly upregulated by epithelial cells upon TLR stimulation or tissue damage^19,20,26,27^. Upon binding to its specific receptor IL-1Rrp2 and recruiting IL-1RAcP, IL-36 initiates downstream NF-κB signaling, driving the transcription and secretion of various pro-inflammatory cytokines, thereby playing a critical role in inflammation and immune regulation^28,29^. Studies have shown that IL-36 is involved in the pathogenesis of multiple inflammatory diseases: in inflammatory bowel disease, it promotes disease progression by inducing CXC chemokines^30^; in generalized pustular psoriasis (GPP), aberrant activation of IL-36 mediates neutrophil chemotactic factor expression, inflammatory cell infiltration, and pustule formation^31^; in chronic rhinosinusitis, IL-36 is regulated by TLR signaling, impairing endothelial barrier function and exacerbating inflammation ^32^; in patients with Pseudomonas aeruginosa-induced ARDS, levels of IL-36γ in plasma and BALF are significantly elevated compared to healthy controls^20^. In ARDS models, IL-36 promotes NETosis and neutrophilic inflammation, a function that parallels the pro-inflammatory actions of IL-1β and IL-18 in acute lung injury, yet the effect of IL-36 appears more localized to the pulmonary epithelium and neutrophils, consistent with its emerging role as an upstream “alarmin” at barrier surfaces^25^. Furthermore, IL-36α promotes the release of pro-inflammatory mediators and T-cell proliferation by activating the NF-κB pathway in macrophages^22^, and enhances the activation of neutrophils, macrophages^22^, and fibroblasts^24,33^. Studies in Jurkat T cells have demonstrated that IL-36 induces mitochondrial activation of MAPK and NF-κB pathways, aggravating inflammatory responses^34,35^. The ability of IL-36Ra to partially reverse lung injury highlights the specificity of IL-36 signaling, analogous to the protective effect of IL-1Ra in IL-1-driven inflammation. These comparisons position IL-36 as a distinct but complementary mediator within the IL-1 family, particularly in the context of sterile neutrophilic lung inflammation^36^. Nevertheless, the precise mechanisms of action remain incompletely understood.

This study demonstrates that in a LPS-induced ARDS model, elevated IL-36 expression is closely associated with aggravated pulmonary inflammation, consistent with previous reports. Specifically, there was a significant increase in the lung wet/dry (W/D) ratio, exacerbated histopathological injury, and markedly elevated levels of inflammatory cytokines (including IL-10 and TNF-α) and MPO in serum and BALF, along with significantly increased expression of corresponding inflammatory proteins in tissues. These results suggest that in sepsis-associated ARDS, IL-36 may exert a pro-inflammatory role potentially through activation of the NF-κB signaling pathway.During the onset of ARDS, large numbers of neutrophils are recruited to lung tissue and generate neutrophil extracellular traps(NETs) ^37^. NETs are complexes capable of trapping pathogens, primarily composed of nuclear chromatin, mitochondrial DNA, and neutrophil granular proteins^38^. They immobilize and eliminate invading pathogens, exert antimicrobial effects, and promote inflammation resolution, constituting an innate immune response that plays a crucial role in host defense ^39^. However, excessive NET formation can damage vascular endothelial cells, promote thrombosis, cause microvascular dysfunction, and injure pulmonary cells, playing a significant role in LPS-induced ARDS ^40,41^. In this study, NETs were induced in neutrophils by PMA stimulation, and their activity was verified using trypan blue staining, establishing an in vitro ARDS model. Consistent with our in vivo findings, immunofluorescence analysis demonstrated that IL-36 priming enhanced the PMA-induced co-localization of MPO and CitH3, indicative of elevated NETs formation. Furthermore, it was observed that IL-36 (α/β/γ) increased the expression of inflammatory cytokines IL-10 and TNF-α, and elevated levels of these cytokines were also detected in the cell supernatant, further confirming that IL-36 induces NETs to secrete substantial pro-inflammatory factors.

The NF-κB signaling pathway is a key transcription factor extensively involved in various biological processes including cellular regulation, inflammatory responses, and immune reactions^42^ . Research indicates that the NF-κB pathway plays an important role in the formation of NETs ^43^ Neutrophils contain dimers composed of NF-κB1 (p50) and p65 NF-κB, and cellular stimulation can trigger nuclear translocation of NF-κB/Rel proteins and degradation of IκB-α^44^. Studies have shown that disruption of NETs can promote apoptosis in gastric cancer cells by modulating the expression of Bcl-2, Bax, and NF-κB ^45^. Conversely, NETs can also enhance NF-κB activation, thereby facilitating the progression and metastasis of breast tumors ^46^. NETs have also been reported to participate in various pathophysiological processes such as wound healing, atherosclerosis, and acute lung injury ^47–50^. This study found that in NET-induced ARDS, IL-36 led to increased protein expression of NF-κB, suggesting that IL-36 may regulate the NF-κB signaling pathway via NETs, activating NF-κB to promote massive secretion of inflammatory factors and resulting in pulmonary injury in ARDS.

In summary, the findings of this study indicate that in LPS-induced ARDS, IL-36 recruits large numbers of neutrophils via NETs, leading to substantial secretion of inflammatory factors and aggravated pulmonary inflammatory injury, while simultaneously activating the NF-κB signaling pathway to further promote extensive release of inflammatory mediators. However, this study utilized an LPS-induced ARDS model, which, while informative for innate immune activation, does not fully replicate the complexity of polymicrobial sepsis. Thus, the findings should be interpreted as preliminary insights into the role of IL-36 in neutrophil-driven lung injury, and further validation in clinically relevant sepsis models is warranted. several notable limitations must be acknowledged, including a limited sample size and the absence of independent clinical validation. Furthermore, long-term outcome data are currently unavailable. Future studies should prioritize mechanistic investigations—such as utilizing IL-36 knockout models-alongside clinical translation trials and comprehensive long-term safety assessments.

## Supplementary Information

Supplementary Information 1.

Supplementary Information 2.

Supplementary Information 3.

Supplementary Information 4.

Supplementary Information 5.

Supplementary Information 6.

Supplementary Information 7.

Supplementary Information 8.

Supplementary Information 9.

Supplementary Information 10.

Supplementary Information 11.

Supplementary Information 12.

Supplementary Information 13.

Supplementary Information 14.

Supplementary Information 15.

Supplementary Information 16.

Supplementary Information 17.

Supplementary Information 18.

Supplementary Information 19.

Supplementary Information 20.

## Data Availability

The datasets used and/or analysed during the current study are available from the first author on reasonable request.
