# Supplementary figures and images for: IL-36 activates neutrophil extracellular traps and exacerbates LPS-induced ARDS in mice
Source: Sci Rep. 2026 May 9;16:21231. doi: 10.1038/s41598-026-51329-w (PMC13347007; doi:10.1038/s41598-026-51329-w)

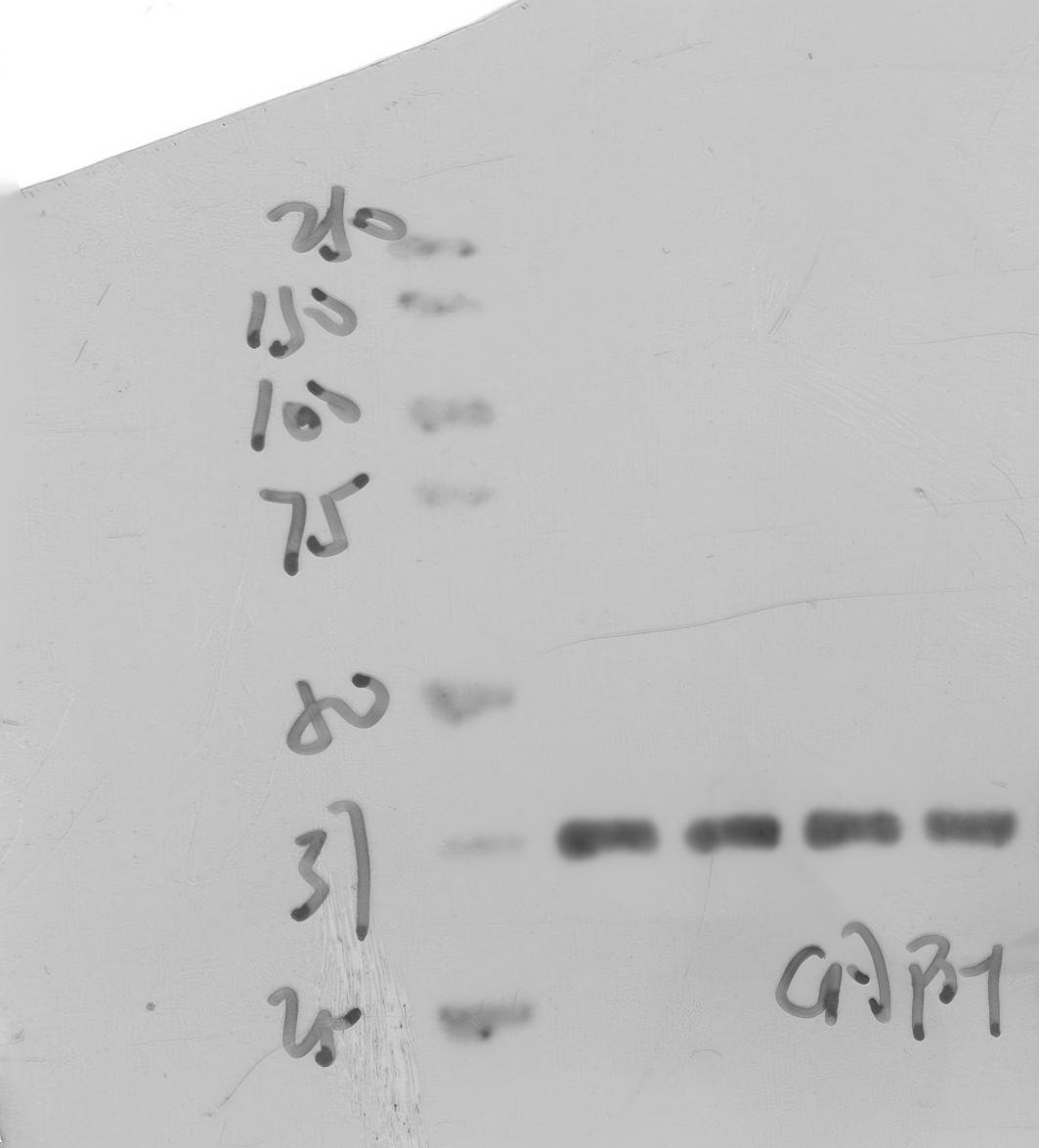

Supplement: Supplementary file 1 — Supplementary Information 1. [file 41598_2026_51329_MOESM1_ESM.jpg]

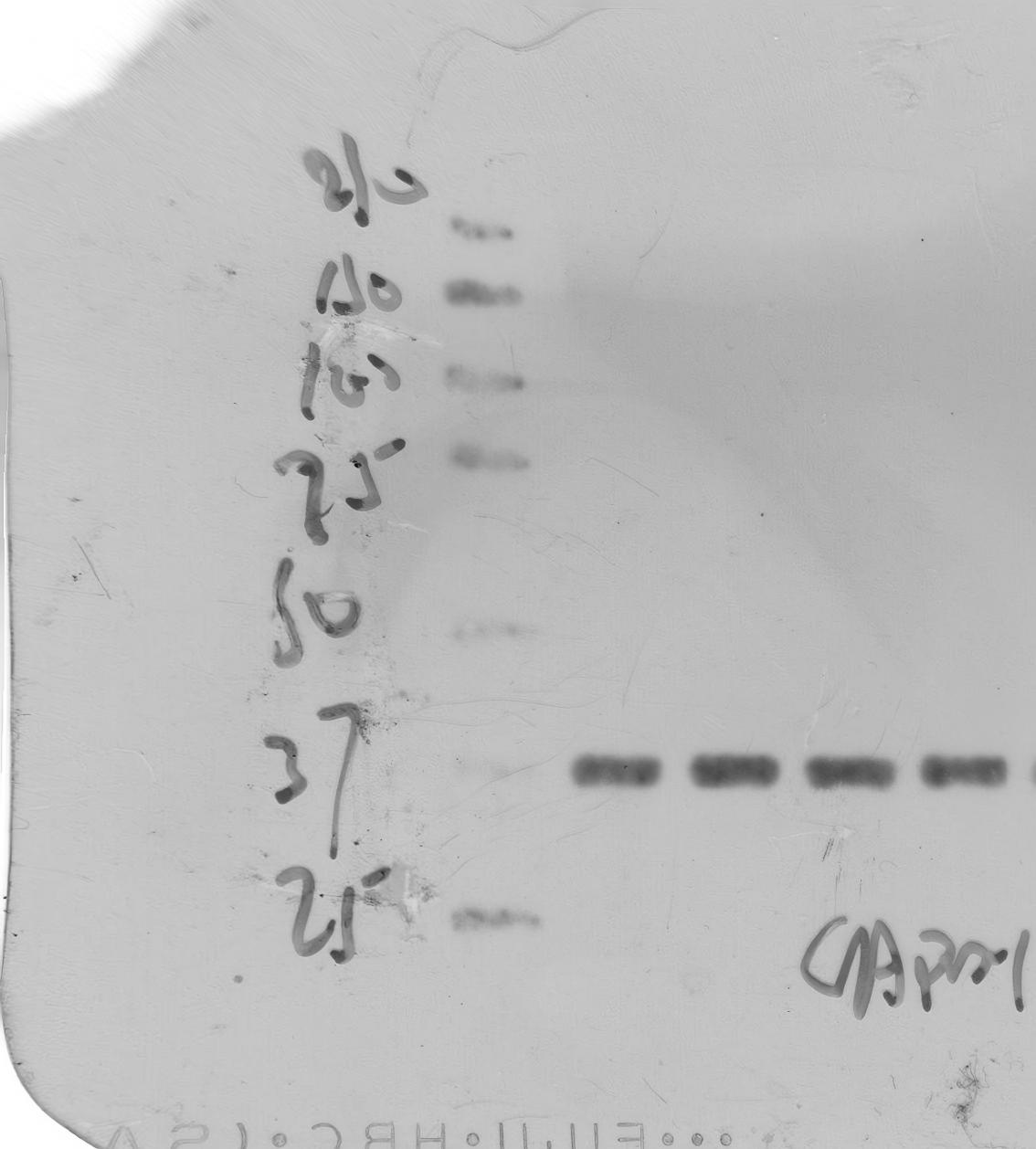

Supplement: Supplementary file 2 — Supplementary Information 2. [file 41598_2026_51329_MOESM2_ESM.jpg]

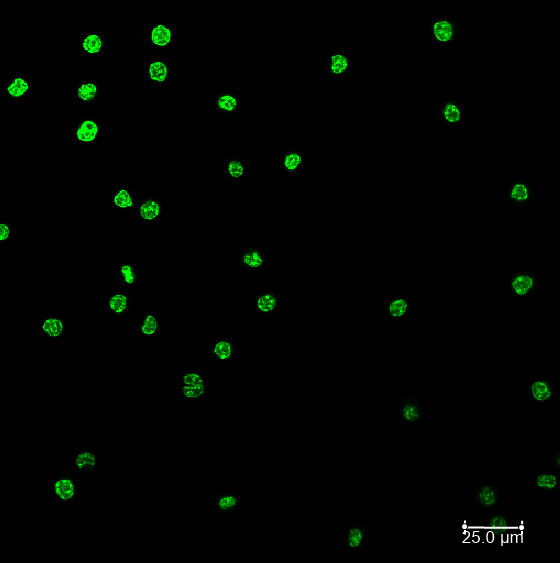

Supplement: Supplementary file 3 — Supplementary Information 3. [file 41598_2026_51329_MOESM3_ESM.tif]

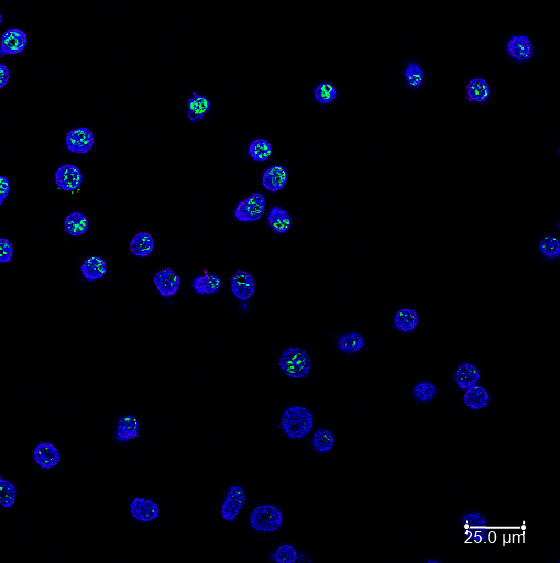

Supplement: Supplementary file 4 — Supplementary Information 4. [file 41598_2026_51329_MOESM4_ESM.tif]

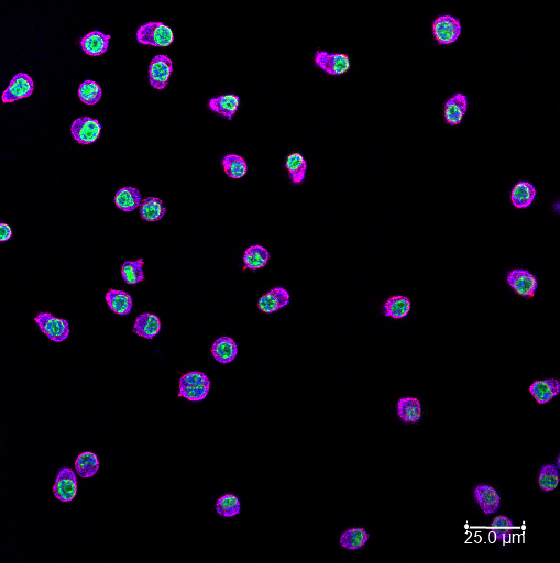

Supplement: Supplementary file 5 — Supplementary Information 5. [file 41598_2026_51329_MOESM5_ESM.tif]

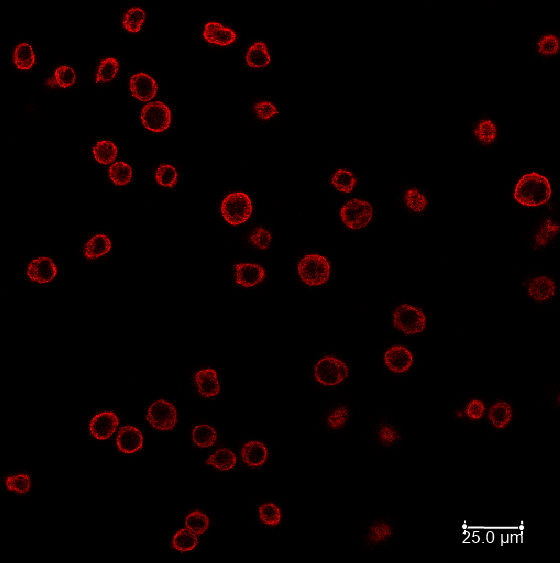

Supplement: Supplementary file 6 — Supplementary Information 6. [file 41598_2026_51329_MOESM6_ESM.tif]

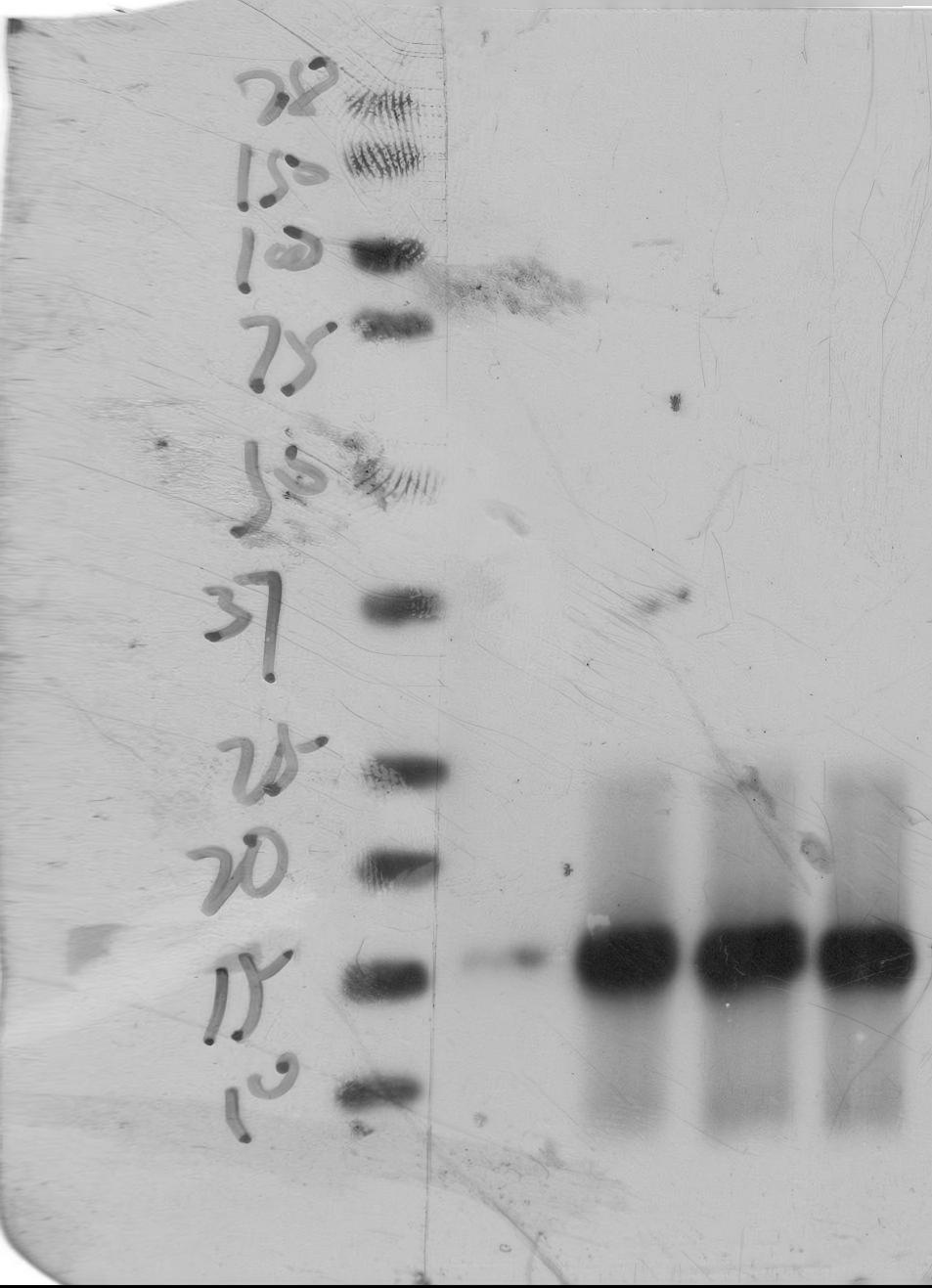

Supplement: Supplementary file 7 — Supplementary Information 7. [file 41598_2026_51329_MOESM7_ESM.jpg]

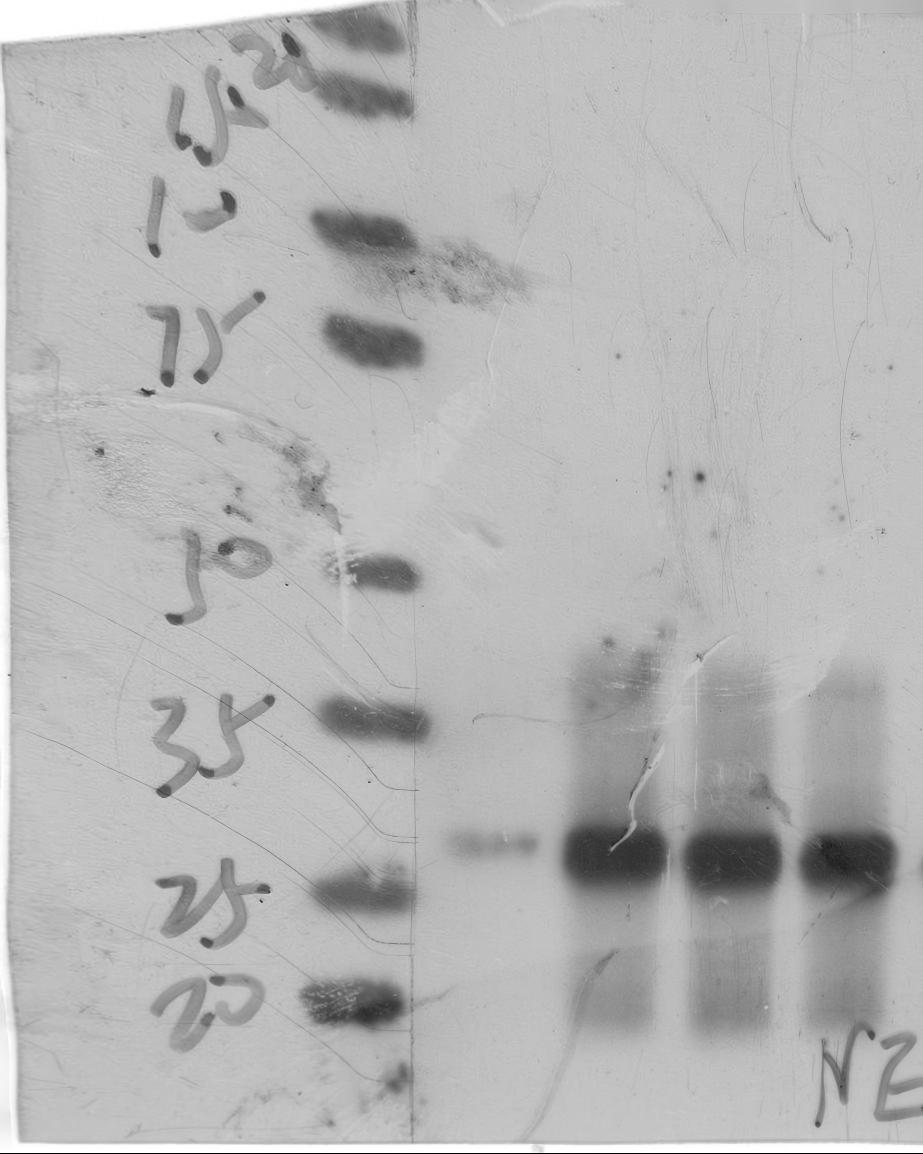

Supplement: Supplementary file 8 — Supplementary Information 8. [file 41598_2026_51329_MOESM8_ESM.jpg]

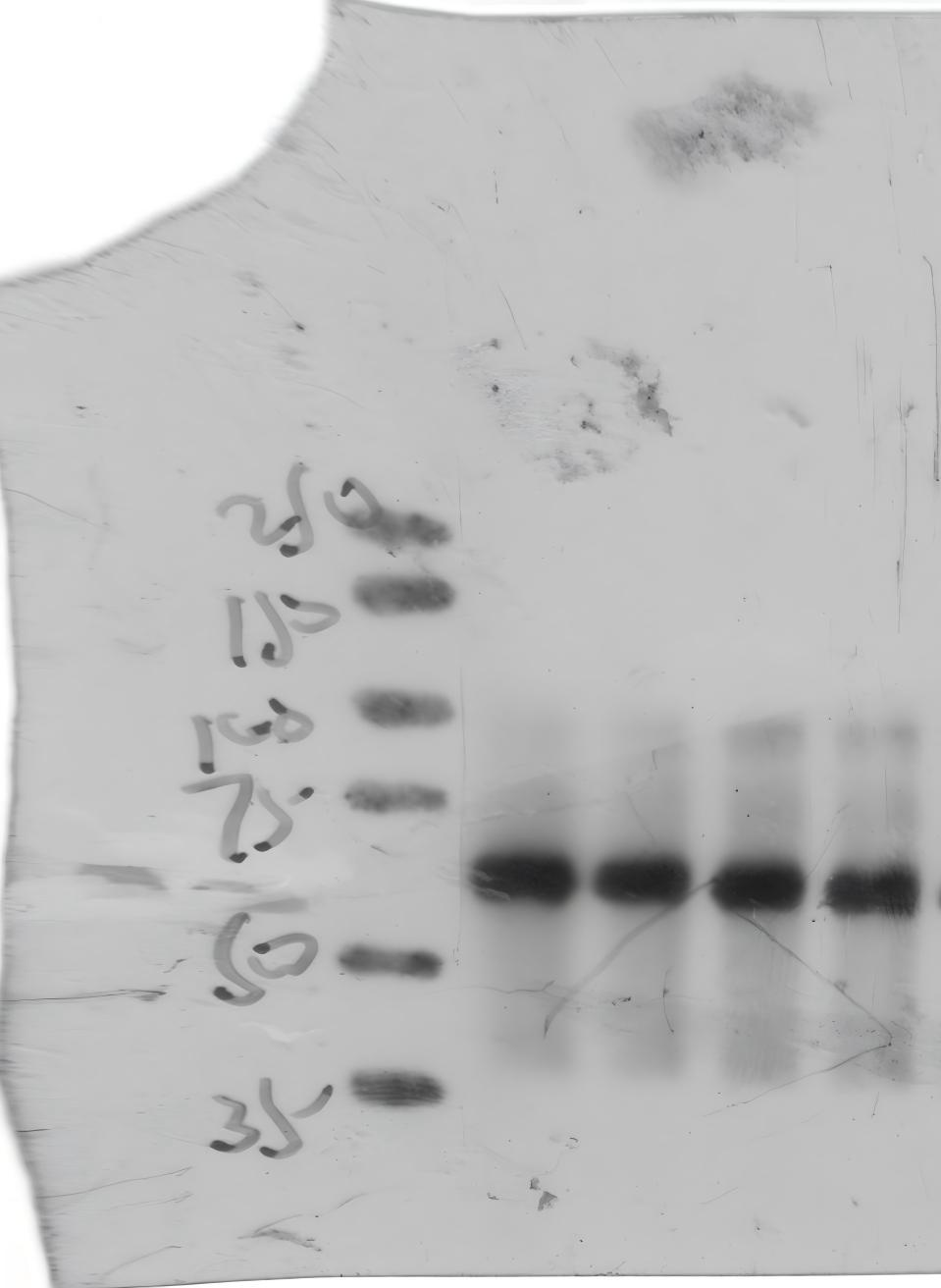

Supplement: Supplementary file 9 — Supplementary Information 9. [file 41598_2026_51329_MOESM9_ESM.jpg]

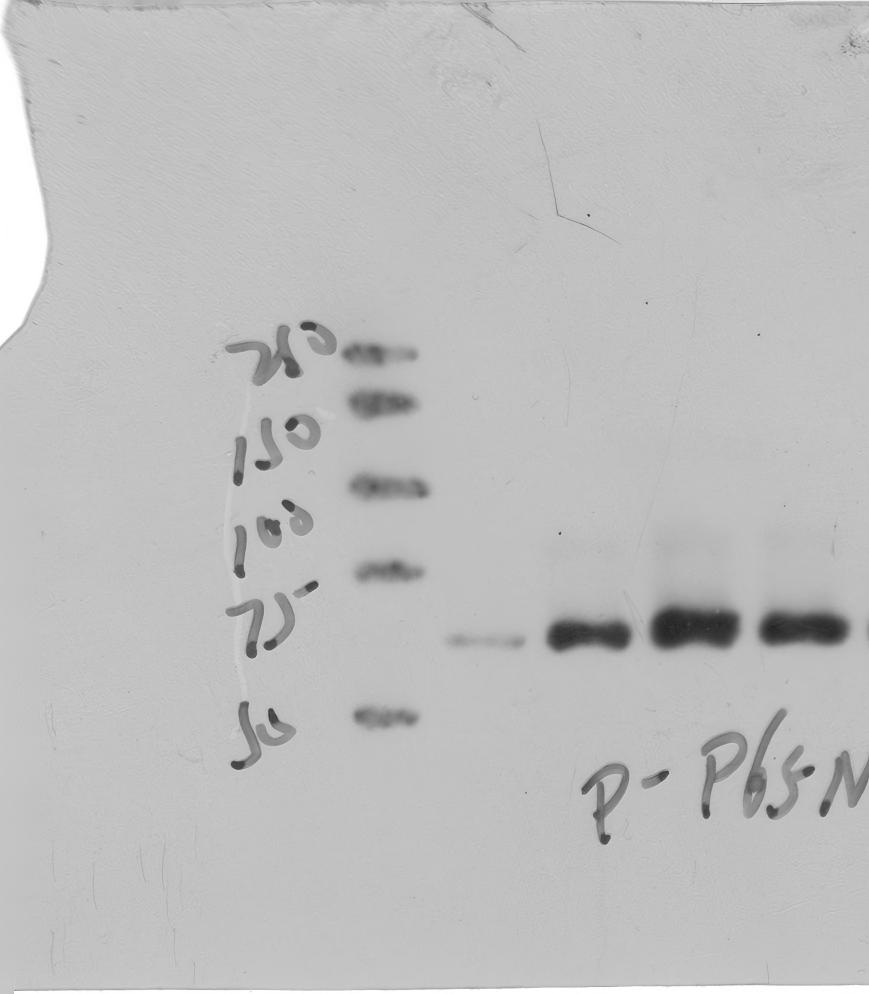

Supplement: Supplementary file 10 — Supplementary Information 10. [file 41598_2026_51329_MOESM10_ESM.jpg]

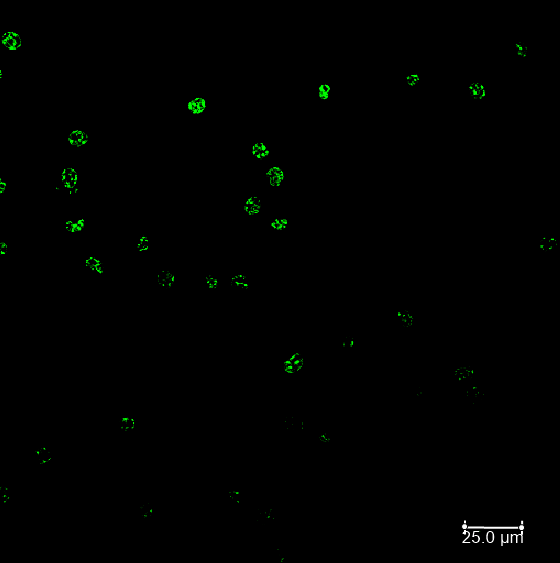

Supplement: Supplementary file 14 — Supplementary Information 14. [file 41598_2026_51329_MOESM14_ESM.tif]

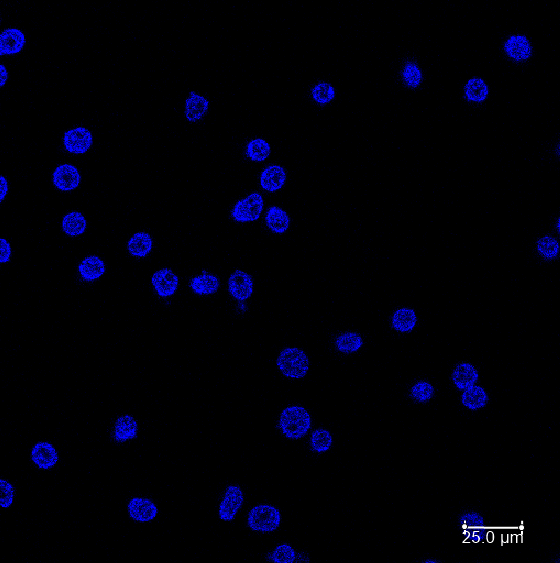

Supplement: Supplementary file 15 — Supplementary Information 15. [file 41598_2026_51329_MOESM15_ESM.tif]

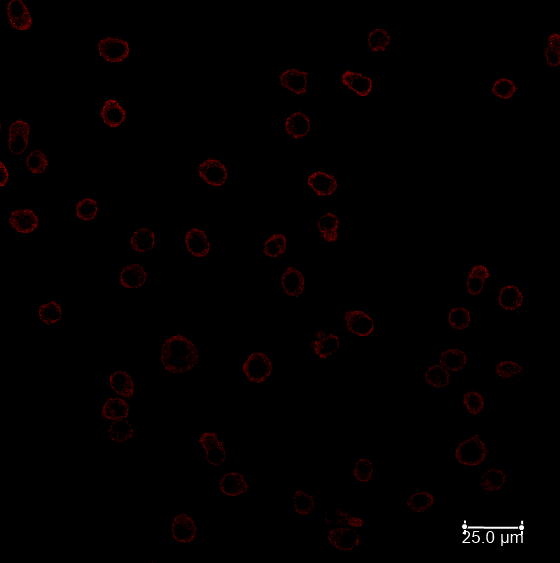

Supplement: Supplementary file 17 — Supplementary Information 17. [file 41598_2026_51329_MOESM17_ESM.tif]
