# Supplementary material for: IL-36 activates neutrophil extracellular traps and exacerbates LPS-induced ARDS in mice
Source: Sci Rep. 2026 May 9;16:21231. doi: 10.1038/s41598-026-51329-w (PMC13347007; doi:10.1038/s41598-026-51329-w)
Supplement: Supplementary file 12 — Supplementary Information 12. [file 41598_2026_51329_MOESM12_ESM.pdf]

姓名：何丽琴　性别：女　年龄：62岁　病区：EICU病区　床号：3　住院号：93251896

2024-04-05，22:31　中性粒细胞胞外诱捕网在脓毒症急性肺损伤小鼠中的作用及其机制的研究

研究项目知情同意书

项目名称：中性粒细胞胞外诱捕网在脓毒症急性肺损伤小鼠中的作用及其机制的研究

方案版本号及版本日期：Version1.0，2024年2月25日

知情同意书版本号及版本日期：Version1.0，2024年2月25日

尊敬的受试者：

我们邀请您参加浙江省人民医院批准开展的（中性粒细胞胞外诱捕网在脓毒症急性肺损伤小鼠中的作用及其机制的研究）研究。本研究将在浙江省人民医院开展，预计将有5名受试者自愿参加。本研究已经得到浙江省人民医院伦理委员会的审查和批准。

本须知将提供给您一些信息以帮助您决定是否参加此项临床研究，您是否参加本项研究完全是自愿的，且您的决定将不会影响到您在本院的正常诊疗权益和待遇。若您选择参加本研究，我们研究团队将在研究过程中尽力保证您的安全和权益！

本须知提供给您一些信息以帮助您决定是否参加此项临床研究。请您仔细阅读，如有任何疑问请向负责该项研究的研究者提出。

一、研究目的：背景意义

脓毒症是严重感染引发的全身炎症反应综合症，至今死亡率居高不下。中性粒细胞的激活对脓毒症急性肺损伤的发展非常重要，而由中性粒细胞释放的中性粒细胞胞外诱捕网在急性肺损伤/急性呼吸窘迫综合征的致病机制中发挥重要作用。

二、研究过程：

本研究为基础研究，随机选取5名患者，采取静脉血标本约2ml，采用Polymorph密度离心法，分离外周血中性粒细胞，进行体外实验。

三、可能的风险与不适

对于您来说，所有的信息将是保密的。您的样本采集为常规静脉抽血，我们将严格按照无菌要求操作，不存在相关风险。

四、预期获益：

通过对您的标本进行预处理，期望能为脓毒症的研究提供有益的信息。

五、替代治疗：

本研究为基础性研究，不涉及治疗的干预。

六、免费治疗：

研究仅采取患者外周血，费用均为课题经费支出，无需患者支付。

七、赔偿：

本研究为基础性性研究，为体外实验，不涉及和研究相关的损害。

八、保密性：

任何研究中获得的关于您的信息将被存储在浙江省人民医院急诊医学科，科研数据硬盘中并被严格保密，且仅用于本研究。任何有关本项研究结果的公开报告不会披露您的个人身份信息。我们将在法律允许的范围内，尽一切努力保护您个人医疗资料的隐私。

在必要情况下，研究者、研究主管部门、伦理委员会及上级核查部门将在签署保密协议的前提下被允许查阅您的医疗记录及相关信息。当您签署了这份知情同意书，代表您同意您的个人和医疗信息被用于上述所描述的场合。

九、自愿性：

您可以选择不参加本项研究，或者在任何时候通知研究者要求退出研究，您的数据将不纳入研究结果，您的任何医疗待遇与权益不会因此而受到影响。

如果您需要其它治疗，或者您没有遵守研究计划，或者发生了与研究相关的损伤或者有任何其它原因，研究医师可以终止您继续参与本项研究。

十、受试者义务：

作为研究受试者，您有以下职责：如实提供有关自身病史和当前身体状况的真实情况；告诉研究医生自己在本次研究期间所出现的任何不适；不得服用受限制的药物、食物等；告诉研究医生自己在最近是否曾参与其他研究，或目前正参与其他研究。

十一、联系方式：

您可随时了解与本研究有关的信息资料和研究进展，若发生与本研究相关的安全性新信息，我们也会及时通知您。如果您有与本研究有关的问题，或您在研究过程中发生了任何不适与损伤，或有关于本项研究参加者权益方面的问题您可以通过85893634与李声琴联系。

如果您对参与本研究的权益有任何问题或诉求，您可以联系浙江省人民医院伦理委员会，联系电话：0571-85893643。

我已经阅读了上述有关本研究的介绍，且研究医生已向我详细地讲解了研究内容，在签署知情同意书前我

姓名：何丽琴　　性别：女　年龄：62岁　　病区：EICU病区　　床号：3　住院号：93251896

已没有更多有关研究的疑惑需咨询。在此基础上，我自愿参加本文所介绍的临床研究，并且我的决定是基于对参加本研究可能产生的风险和受益充分了解。此外，研究者没有对我使用欺骗、利诱、胁迫等手段强行让我同意参加研究，并且我知道我可以在任何阶段无条件退出研究。

该名受试者因无行为能力、限制行为能力，本知情同意由其监护人或者法定代理人代为签署。

家属签名：鲁[illegible]清 2024年4月5日 23:34

我已准确地将这份文件告知受试者，他/她准确地阅读了这份知情同意书，并有机会提出问题。

研究者姓名：李声琴

医师签名：李声琴　　日期：　2024年04月05日 22:29

仁爱 | 卓越 | 奉献 | 创新
Love Excellence Dedication Innovation
